# Homozygosity Mapping on a Single Patient—Identification of Homozygous Regions of Recent Common Ancestry by Using Population Data

**DOI:** 10.1002/humu.21432

**Published:** 2011-01-25

**Authors:** Lu Zhang, Wanling Yang, Dingge Ying, Stacey S Cherny, Friedhelm Hildebrandt, Pak Chung Sham, Yu Lung Lau

**Affiliations:** 1 Department of Paediatrics and Adolescent Medicine, University of Hong KongHong Kong; 2 Department of Psychiatry and the State Key Laboratory for Brain and Cognitive Sciences, University of Hong KongHong Kong; 3 Department of Pediatrics, University of Michigan School of MedicineAnn Arbor, Michigan; 4 Department of Human Genetics, University of Michigan School of MedicineAnn Arbor, Michigan; 5 Howard Hughes Medical Institute, University of Michigan School of MedicineAnn Arbor, Michigan

**Keywords:** homozygosity mapping, recessive mutation, founder mutation, rare variants, population-based linkage

## Abstract

Homozygosity mapping has played an important role in detecting recessive mutations using families of consanguineous marriages. However, detection of regions identical and homozygosity by descent (HBD) when family data are not available, or when relationships are unknown, is still a challenge. Making use of population data from high-density SNP genotyping may allow detection of regions HBD from recent common founders in singleton patients without genealogy information. We report a novel algorithm that detects such regions by estimating the population haplotype frequencies (*HF*) for an entire homozygous region. We also developed a simulation method to evaluate the probability of HBD and linkage to disease for a homozygous region by examining the best regions in unaffected controls from the host population. The method can be applied to diseases of Mendelian inheritance but can also be extended to complex diseases to detect rare founder mutations that affect a very small number of patients using either multiplex families or sporadic cases. Testing of the method on both real cases (singleton affected) and simulated data demonstrated its superb sensitivity and robustness under genetic heterogeneity. Hum Mutat 32:345–353, 2011. © 2011 Wiley-Liss, Inc.

## Introduction

Autosomal recessive mutations are involved in Mendelian diseases and probably a small proportion of cases of complex diseases. Detecting such mutations holds much promise in improving our understanding of disease mechanism and gene function. The power of detection is lower for recessive mutations in most situations compared to autosomal dominant ones, because usually the number of affected is small in a family. Also, genetic heterogeneity often poses problems even when multiple families for a disease are available. Homozygosity mapping was designed to increase the power of detection for recessive mutations by recognizing that a proportion of such families are inbred, and these inbred families are, in fact, informative even in the absence of affected siblings [Kruglyak et al., 1995; Lander and Botstein, 1987]. However, the method has been mainly applied to families with apparent consanguineous marriages of close relatives. In many situations, the relationship between parents can be remote and unknown, and the common ancestor is untraceable. Detection of such regions of recent common ancestry requires development of novel methods.

The challenge in detecting such recessive mutations is how to distinguish the haplotypes that are likely derived from a recent founder (homozygosity by descent (HBD), defined by 5–50 generations of recombination events, for example) from those that are more likely to have arisen within a population that is defined by hundreds or thousands of generations of recombination events (homozygosity by chance, HBC). Although the length of the homozygous regions has been used to help with this distinction and in mutation detection [Carr et al., 2006, 2009], numerous studies have shown that length alone is a poor parameter in revealing a region's history [Gibson et al., 2006; Lencz et al., 2007; Li et al., 2006; McQuillan et al., 2008].

Traditional homozygosity mapping is based on inference of HBD using a relatively sparse marker set (typically 400–800 microsatellite markers). This means that the detection is relatively low resolution and often relies on having genotype data on family members. Furthermore, linkage disequilibrium (LD) among markers on a population scale is often irrelevant for this level of marker density. Moreover, none of the traditional linkage analysis tools can deal with distant relationships that are usually unclear in modern societies, unless markers of extremely low population allele frequency are typed and closer relationships are assumed to make analysis computationally feasible. Although these programs make use of population marker allele frequency, without being able to use LD information, there is a limitation in estimating haplotype frequencies (*HF*) even with multipoint analysis and dense single nucleotide polymorphism (SNP) marker genotyping. Making full use of high-density SNP genotyping and haplotype frequency (i.e., LD) information derived from population data or reference databases (such as HapMap), it becomes possible to construct a method with greater power and resolution in identifying ancestral haplotypes, which requires neither pedigree structure information nor genotype data on family members (e.g., [Sham et al., 2009]).

In the present study, we developed an algorithm to estimate the *HF* of any homozygous region in a patient's genome, making use of information from unaffected individuals from the same population rather than relying on data from family members. This approach extends the conceptual framework of homozygosity mapping and the pioneer work by Houwen et al. [1994] to apply to high-density SNP genotyping data and to distantly related patients even when the relationship is unknown.

The accompanying software implementing the algorithm, Homozygous Regions of Recent Ancestry (HRRA), can be used with recessive Mendelian diseases when only one or a few patients are available and with no genealogy data. Rare, recessive founder mutations may also play a role in patients with severe manifestations of complex diseases, or complex traits at the extreme tail of the population distribution. Both linkage and association studies for such situations are constrained by power and genetic heterogeneity. HRRA can be extended to such situations to identify recent founder mutations even if the mutation may affect only a very small number of patients and the genetic variants have very low population frequency.

## Materials and Methods

### Estimating the *HF* of a Homozygous Region in a Single Individual

Estimating the *HF* of a homozygous region is a simple and accurate way of distinguishing regions HBD from a recent common ancestral founder from regions HBC. We adopted a Markov model to estimate the *HF* of an entire homozygous region, because directly estimating allele frequency of a long region can be computationally challenging and inaccurate [Kong et al., 2008]. In this method, the population haplotype frequency for two adjacent SNPs is first estimated based on data from population controls, according to an Expectation–Maximization (EM) algorithm [Clark et al., 2001; Fallin and Schork, 2000]. Consider two SNPs A (with alleles *A* and *a*) and B (with alleles *B* and *b*), with allele frequencies designated *P*_*A*_ (*P*_*a*_) and *P*_*B*_ (*P*_*b*_), such that *P*_*A*_+*P_a_*=1, and *P*_*B*_+*P*_*b*_=1. The four haplotypes that can be formed by the two SNPs are *AB*, *Ab*, *aB*, and *ab,* and the *HF* for these haplotypes are designated *P*_*AB*_, *P*_*Ab*_, *P*_*aB*_, *P*_*ab*_, and can be estimated based on the control samples using the EM algorithm. In situations where a particular haplotype (e.g., *AB*) does not appear in the control samples, its *HF* (i.e., *P*_*AB*_) is replaced by *K*, assuming *K* is such that there is a 95% probability that it will not be observed in the controls based on the sample size (*v*):


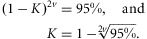


A Markov model is then constructed by using the pairwise *HF* to evaluate the *HF* for an entire homozygous region. Let *G*_*i*_ denote the *i*th SNP in the entire homozygous region, containing SNPs *G*_1_ to *G*_*n*_, and 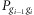 is the pairwise *HF* between alleles 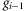 and *g*_*i*_ at adjacent SNPs 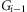 and *G*_*i*_, and 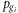 is the frequency for the *g*_*i*_ allele of SNP *G*_*i*_, the *HF* from *G*_1_ to *G*_*n*_ then can be calculated as:


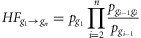


Due to the homozygous nature of the region, the probability of this region appearing by chance in this population can be defined as *HBC*_*s*_=*HF*^2^, with the subscript “s” indicating a single-patient scenario.

### Evaluating Situations Where Multiple Individuals Share a Common Homozygous Allele

The following algorithm is adopted to evaluate situations where multiple individuals share a common homozygous region. *HF*^2^ as described above is used to evaluate a homozygous region in a single individual. Let us assume that the number of patients sharing the same homozygous region is *N* and the total number of patients being considered is *T*. A parameter *HBC*_*m*_ is introduced here representing the probability of the region being shared by *N* individuals:


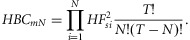


For example, when four patients share a common homozygous region in a total of 300 patients, *HBC*_*m*_ can be calculated as:


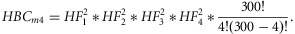


### Estimating the Probability of HBC Through a Simulation Process

Although *HBC*_*s*_ and *HBC*_*m*_ reflect in a way the random chance for a homozygous region to appear or to be shared in controls, as with nominal *P*-values in genome-wide studies, direct evaluation of statistical significance based on these parameters is difficult. For example, uneven coverage across the genome may make these parameters noncomparable among different regions. Therefore, we used a simulation method to try to derive a corrected genome-wide significance measure.

For the simulation process for a single-patient scenario, the homozygous region with the smallest *HF*^2^ in the entire genome in each control individual is recorded, and these frequencies provide a null distribution for assessing the empirical, genome-wide probablility for a homozygous region to appear by chance in a particular patient. By so doing, we measure each homozygous region against the best regions in the genomes of controls and therefore reduce false positive detections. This also helps overcome uneven coverage of different regions of the genome as only the region with the best *HF*^2^ contributes to this null distribution.

In the situation where multiple patients (*N*) from a pool of a total of *T* patients share a common homozygous region, the simulation was performed such that each time *T* individuals are randomly selected from all the available control samples, and the common homozygous region shared by *N* or fewer individuals that produces the best *HBC*_*mN*_ value is recorded for each simulation. Afterward, the region with the best *HBC*_*mN*_ in this round of simulation will be excluded from further consideration. The *HBC*_*mN*_ parameters generated through thousands of simulations are used to form the null distribution of this parameter. Again, the area under the curve on the right is used to estimate the probability of a homozygous region shared by *N* out of *T* patients by random chance.

When evaluating shared homozygous regions for affected sib pairs, within-family homogeneity is assumed. The region that produces the smaller *HF*^2^ between two siblings in each family is used for the calculation of *HBC*_*m*_, a between-family parameter. Because the chance for a sib pair to share two alleles identical by descent (IBD) is one-quarter, (1/4)*^N^* is also factored into the calculation of the *P*-values afterward, and *N* stands for the number of sib pairs who share the same founder homozygous allele.

### Simulation of Founder Alleles and the Inheritance Process

**Figure 1:**
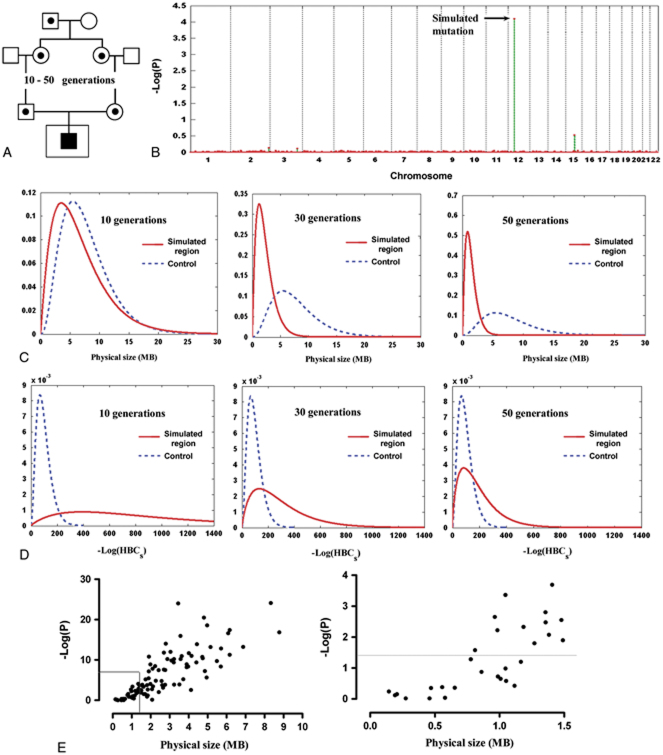
HRRA results on singleton patient cases. **A:** The simulation process. Shown is the process of generating the genotypes of the “affected” individual who inherits two copies of a recent ancestral allele. The data made available to HRRA are the genotypes of the singleton “affected” individual (inside the larger square) plus genotype data for control individuals. **B:** HRRA result on a representative simulated case. This case inherited two copies of a haplotype that is 30 generations in age and the *P*-value for this region ranks at the 50th percentile among all simulations. The *y*-axis is the −log of *P*-values generated through a simulation process and the *x*-axis is the chromosomal position. **C, D:** The distributions of the simulated homozygous regions and the best homozygous regions in the controls based on their physical length (C) or the −log of *HBC*_*s*_ estimated by HRRA (D). The *x*-axis is MB (C) or −log(*HBC*_*s*_) (D) of the simulated haplotypes (solid line) aged 10 generations (left), 30 generations (middle), or 50 generations (right), and the best regions in the control individuals (dashed line). Calculation of the *y*-axis is based on a probability density function (gamma distribution) of the physical length (MB) or −log(*HBC*_*s*_) of the regions considered (see Supporting Information). **E:** Correlation between physical size and HRRA *P*-values. Left panel: overall correlation (*R*^2^=0.645); right panel: zoomed in on regions 1.5 MB and smaller. [Color figures can be viewed in the online issue, which is available at http://www.wiley.com/humanmutation.]

The simulation process is depicted in Figure 1A, which is similar to one we described previously [Yang et al., 2008]. Briefly, a mutation was assigned randomly to one of the chromosomes of an individual serving as an ancestor, and meiosis events of between 10 and 50 generations were then simulated, shortening the mutation-carrying haplotype further and further by recombination events. Afterward, an “affected” individual was simulated to inherit two copies of the ancestral allele, generated through two independent inheritance paths. Detailed description of the simulation process can be found in the Supporting Information. All the simulations used data on Hong Kong Chinese genotyped by Illumina 610-Quad as reported previously [Yang et al., 2010].

### Real Cases

Six patients with an autosomal recessive kidney disease, nephronophthisis (NPHP), who are known to carry homozygous mutations in one of the 13 candidate genes, were used to test our program (F30-2, F399-1, F408, F409, A159 A1730-2; from [Hildebrandt et al., 2009]). The samples were genotyped using the Affymetrix Human Mapping 250K StyI Array platform. Controls were 112 nonfounders from HapMap Phase II CEPH data. There were 180,000 overlapping SNPs between the HapMap data and the 250K StyI platform, which were used in this test.

## Results

### Detection of Homozygous Regions of Recent Ancestry in a Singleton Patient

In Figure 1B, we showed the result evaluating a representative case carrying a simulated homozygous region HBD of 30 generations in age. The *HF*^2^ for this region was calculated as described in Materials and Methods and the *P*-value for this parameter was estimated according to the null distribution derived from the best regions in the control individuals. The *P*-value for this HBD region in this case ranked at the 50th percentile among all the simulations. It is clear that the simulated region can be easily distinguished from other homozygous regions in this individual's genome. We also compared evaluating the simulated homozygous regions by either their physical length (Fig. 1C) or their *HF*^2^ (Fig. 1D), in contrast to regions assumed HBC in controls. The results clearly demonstrated the superiority of *HF*^2^ in evaluating regions derived from recent common ancestry compared to evaluations based on physical size of the homozygous regions.

It is clear that for haplotypes that have gone through 10 generations of meioses, the simulated homozygous regions can be easily distinguished from the best regions in the control individuals in most cases (Fig. 1D, left panel). For haplotypes that have gone through 30 generations of meioses or more, only in certain cases can they be distinguished from those in controls (Fig. 1D, middle and right panels), although the separation would also depend on the consanguinity of the host population and the population history (the older and the less consanguineous the population, the better the detection in terms of the age of the recent common ancestry).

Analysis of the correlation between physical sizes of the regions HBD and their corrected *P*-values evaluated by HRRA revealed a correlation coefficient of 0.645. As seen in Figure 1E, regions significantly smaller than 0.7 MB are basically undetectable and regions larger than 2 MB can be easily detected in most cases, with the detectability varying for regions in between those sizes.

We examined how the modern linkage analysis software Merlin [Abecasis et al., 2002] would perform in detecting these simulated homozygous regions. In order to make Merlin work with these situations which it is not designed to handle, we assumed a second cousin marriage and added an affected sibling with no genotype data, a method similar to what Hildebrand et al. [2009] employed (Supp. Fig. S1a). The result from Merlin analyzing the same case as shown in Figure 1B is presented in Supp. Figure S1b. It can be seen that the simulated region does not stand out by physical size, and many other regions in this individual's genome achieved the same LOD score as the simulated homozygous region.

### Detection of Homozygous Regions HBD Shared by Multiple Patients

**Figure 2:**
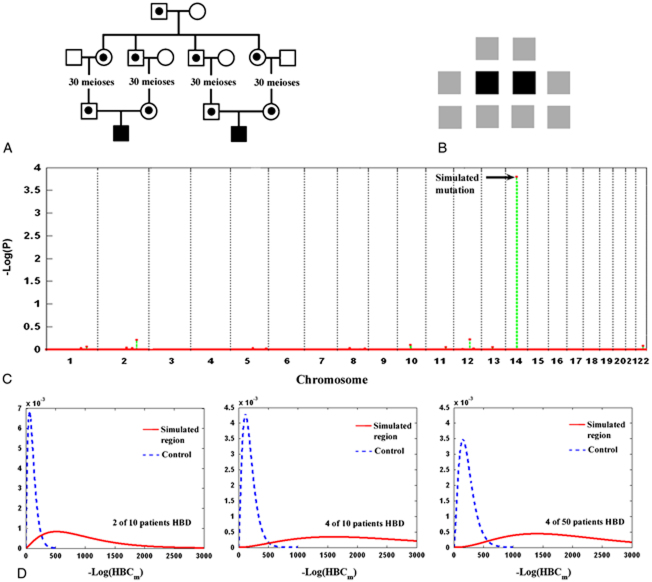
Multiple patients sharing a region HBD. **A:** The simulation process. Two individuals were simulated to each inherit two copies of a common founder mutation allele. **B:** Input data to HRRA. Genotype data on 10 patients (all singleton patients) is made available to HRRA, of which two individuals carry the same homozygous mutation. **C:** Result on a representative simulated case. The *P*-value on the region HBD (30 generations in age) for this case ranked at the 50th percentile in all simulations. **D:** Distribution of the simulated mutation regions (solid line) and the best regions shared by a corresponding number of controls (dashed line). Left: 2 of the 10 patients were simulated to inherit two copies of a recent founder allele and they were analyzed together with eight other patients who do not inherit the allele. Middle: 4 of the 10 patients were simulated to inherit the same founder allele HBD and they were analyzed together with other 6 patients who do not inherit the allele. Right: 4 of a total of 50 patients were simulated to inherit two copies of a recent founder allele and they were analyzed together with another 46 individuals who do not carry the allele. [Color figures can be viewed in the online issue, which is available at http://www.wiley.com/humanmutation.]

The next question we asked was whether additional patients sharing a homozygous region HBD increases the sensitivity of detection, and whether any increased sensitivity would still hold in the face of increased genetic heterogeneity. We simulated a situation in which two patients each inherited two copies of the same recessive mutation (30 generations in age; Fig. 2A). The two individuals were then mixed with eight others (all unrelated individuals) who do not carry the same homozygous haplotype for analysis by HRRA (Fig. 2B). In Figure 2C, we showed the evaluation result on a representative case whose *P*-value for the simulated region ranked at the 50th percentile among all the simulations. The region did stand out, compared to other homozygous regions in these individuals' genome, with a significant *P*-value. In Figure 2D, we showed the separation between the simulated regions and the best homozygous regions in the controls in three situations: 2 in 10 patients, 4 in 10 patients, and 4 in 50 patients sharing the same founder haplotype HBD. The results indicate that increasing the number of patients sharing the same haplotype allele would aid its detection (comparing the middle and the left panels), whereas the total number of patients considered (genetic heterogeneity) had little effect (comparing the right and the middle panels).

### Detection of Regions HBD in Unrelated Cases of Complex Diseases

Even for complex diseases, major mutations may play a role in a small proportion of patients, particularly among those who display unique manifestations. Some of the mutations could be recessive and arose recently in history. Therefore, we tested whether a recessive mutation affecting a very small proportion of patients in a case–control study can still be detected using HRRA.

**Figure 3:**
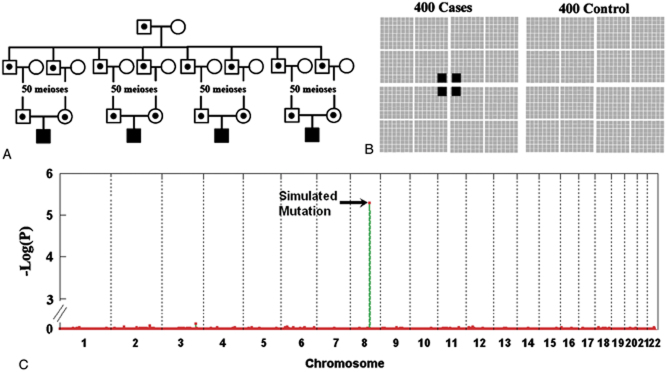
A case–control study. **A:** The simulation process. Four individuals were simulated to have inherited two copies of a common founder mutation allele. **B:** Input to HRRA. These four individuals were mixed with 396 others and they were analyzed by HRRA. All the individuals were assumed to be unrelated. **C:** HRRA result on a representative simulation. Shown is an HRRA result on a representative simulation whose *P*-value on the simulated region ranked at the 50th percentile among all the simulations. [Color figures can be viewed in the online issue, which is available at http://www.wiley.com/humanmutation.]

As shown in Figure 3, we simulated four individuals who each inherited two copies of a common haplotype derived from a recent ancestor (50 generations in age). These four individuals were mixed with 396 other individuals in an assumed case–control study scenario and were examined by HRRA (Fig. 3B). Figure 3C shows the result from a representative simulation for which the HRRA *P*-value ranked at the 50th percentile among all the simulated cases. Similar to scenarios in diseases of Mendelian inheritance, our algorithm is sensitive to the absolute number of individuals who share a common homozygous region and is very much immune to the total number of the patients considered. This is an important feature for detecting rare, recessive mutations that may affect a very small proportion of patients of complex diseases, especially for genome-wide association studies when usually a large number of samples are studied.

### Detection of Regions HBD from Multiplex Families—Sib Pairs as an Example

**Figure 4:**
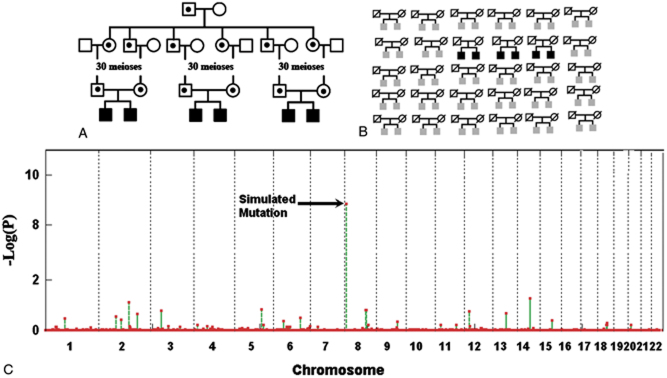
Affected sib pairs. **A:** The simulation process. Three affected sib pairs were simulated to each have inherited two copies of a recent founder mutation allele. **B:** Pedigree input to HRRA. This includes the sibling data on the three pairs who carry the simulated mutation region and 27 other sib pairs who do not carry the mutation region. **C:** HRRA result on a representative simulation. For this case the *P*-value for the simulated region ranked at the 50th percentile among all simulations. [Color figures can be viewed in the online issue, which is available at http://www.wiley.com/humanmutation.]

Linkage on multiplex families has been used extensively in the search for mutations involved in complex diseases, largely by nonparametric methods. Here we examined whether HRRA can detect such a recessive mutation when only a small proportion of multiplex families carries the mutation haplotype. We simulated three sib pairs who inherited the same founder mutation haplotype that is 30 generations in age from a common ancestor (Fig. 4A). The three sib pairs were analyzed together with 27 other sib pairs who do not carry the same haplotype allele (Fig. 4B). Very significant *P*-values were achieved from the majority of simulations, as demonstrated by a representative case whose *P*-value for the simulated region ranked at the 50th percentile among all the simulations (Fig. 4C). The simulated region could not be detected by traditional linkage analysis methods using either a parametric (based on a recessive model; Supp. Fig. S2, left panel) or nonparametric (Supp. Fig. S2, right panel) method [Kong and Cox, 1997].

### Real Case Examples

**Figure 5:**
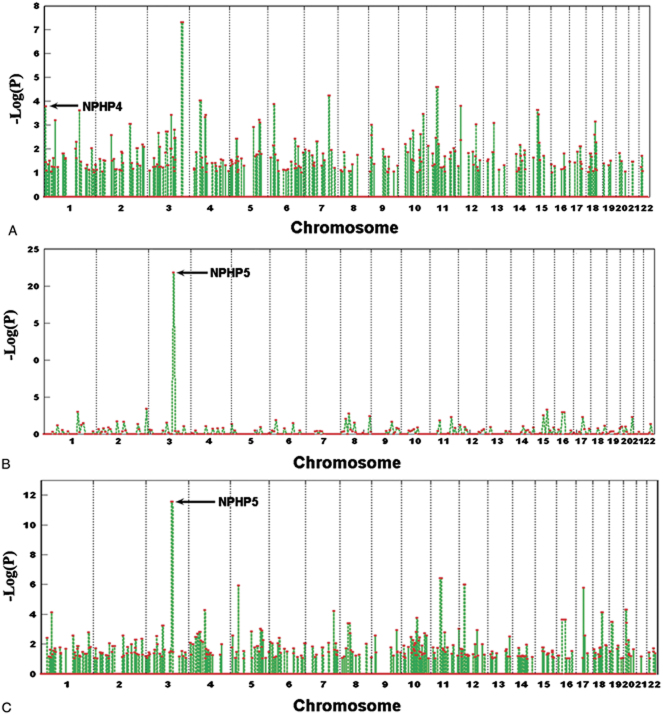
HRRA results on real cases. Shown are the HRRA results on real cases of NPHP disease with known homozygous mutations. The arrows point to the regions containing the known mutated genes. The cases are F30-2 **(A)**, F399-1 **(B)**, and F408 **(C)** as in Hildebrandt et al. 2009. [Color figures can be viewed in the online issue, which is available at http://www.wileycom/humanmutation.]

We evaluated six real cases with nephronophthisis (NPHP) disease, which have known homozygous mutations on one of the 13 candidate genes [Hildebrandt et al., 2009]. For three of the six cases, we detected the mutation region with corrected genome-level *P*-values ranging from 10^−3^ to 10^−25^ (Fig. 5). For patient F30-2 (Fig. 5A), the region where the mutated *NPHP4* gene is located is among a number of regions that had a *P*-value smaller than 10^−3^, which may reflect the age of the mutation region or the consanguinity of the population [Hildebrandt et al., 2009]. The region where the *NPHP5* gene is located showed impresssive *P*-values in two other cases (Fig. 5B and C), indicating that recent founder mutations may have accounted for these cases, although both the two patients are from outbred populations with no known consanguineous marriages.

Three other cases evaluated by HRRA did not show detectable regions HBD in the chromosomal regions where the 13 known genes are located. It is possible that the homozygous regions where the mutations reside may be too short (reflecting longer history), or have poor coverage by the genotyped SNPs (data on only 180,000 markers overlapping the genotyping platform and HapMap are used in this analysis). Although a slight mismatch between the cases (from various Caucasian populations) and the controls (from HapMap CEPH) may partially explain the higher background we saw from the real cases than from the simulations (for which both cases and controls are from the same population), consanguineous marriages in either families or populations (such as the case in Fig. 5A) are probably playing a major role for the higher background seen. Comparison between results from simulation and those from real cases points to the benefit of good marker coverage as well as matching of population background between the cases and reference controls.

## Discussion

Genetics has seen successes in identifying causal mutations when large pedigrees are available, and in identifying common susceptibility alleles to complex diseases with large sample collections. However, detecting the rare variants will continue to remain a challenge until large-scale whole-genome sequencing becomes a reality. Rare variants of relatively large effect size may be enriched in patients of certain manifestations, such as patients with specific subphenotypes, early onset age, or familial aggregation. Some of the rare variants may be relatively new mutations and may affect multiple patients of unknown relationship in a given population. Methodologies for detecting these rare variants without the help of familial data and in the face of genetic heterogeneity are still lacking and may have significant impact on our endeavor in finding disease genes.

Homozygosity mapping has played a vital role in the identification of many recessive causal mutations. Expanding the framework of homozygosity mapping to samples without known genealogy and with limited number of affected individuals (down to a single case) remains a daunting challenge. It is also appealing to extend this framework to multiplex family collections of complex diseases, such as affected sib pairs. Many of the multiplex families have been studied in the late 1990s with limited success, probably due to lack of power and both locus and allelic heterogeneity among families.

Numerous attempts have been made to evaluate homozygous regions in patients in order to detect recessive mutations using high-density SNP genotyping data [Carr et al., 2006; Seelow et al., 2009; Wang et al., 2008]. However, few have explicitly utilized population information, in terms of haplotype frequencies, in their evaluation of homozygous regions. Most programs developed so far are tools that allow visualization of such regions from SNP genotyping data [Carr et al., 2006; Seelow et al., 2009; Woods et al., 2004], which rely on the size of the homozygous regions. As shown in Figure 1, size alone can be a poor parameter in evaluating homozygous regions. Furthermore, numerous studies have pointed out that long tracts of homozygous regions in our genome are common, even in apparently outbred populations [Gibson et al., 2006; Lencz et al., 2007; Li et al., 2006; McQuillan et al., 2008].

Hildebrandt et al. [2009] introduced modifications to the traditional homozygosity mapping method, which allowed detection of certain homozygous regions responsible for autosomal recessive diseases. PLINK [Purcell et al., 2007] is efficient in detecting long haplotypes shared among patients due to shared recent ancestry, but does not explicitly evaluate the probability of HBD versus HBC for the detected regions. Runs of Homozygosity (ROH) [Lencz et al., 2007] is designed particularly for detecting homozygous regions unusually shared among patients compared to controls, but is not designed to detect regions of recent common ancestry. Both methods evaluate haplotype-sharing through counting in cases versus in controls. BEAGLE-IBD [Browning and Browning, 2010] provides sensitive detection of regions HBD, but is limited to detection of pairwise sharing and did not provide a comprehensive evaluation means.

HRRA explicitly uses population information in evaluating homozygous regions through a Monte Carlo simulation process, which not only provides detection of HBD, but also evaluation of a relationship between a region HBD with an underlying disease by calculating the chance of this region appearing in the host population. Different marker density and coverage of rare alleles among different regions in the genome may affect detection sensitivity, and a much better covered homozygous region with more rare markers genotyped may stand out compared to other regions, generating spurious positive results. This is eventually overcome by the simulation process introduced here, which documents the best regions genome-wide in controls as the null distribution of the homozygous regions.

From the results on simulated situations, it is obvious that the total number of individuals sharing a homozygous region IBD is important for detection, rather than the percentage of patients who share the same mutation. Our methodology is able to detect rare variants with allele frequencies of 1% or lower (Fig. 3), indicating that genetic heterogeneity has little effect on mutation detection. This also means that other issues, such as ascertainment bias, misdiagnoses, or phenotypic heterogeneity may have minimal effects on mutation detection by HRRA.

Making full use of population information in terms of allele frequency and LD, the program can actually detect regions smaller than 1 MB in certain cases. The power of detection is increased when (1) the mutation occurred in a region with relatively low recombination rate so that after tens of generations of recombination, a large enough haplotype is still conserved; (2) the haplotype on which the mutation occurred is relatively rare; and (3) the genotyping methodology has good coverage of rare variants to reflect the rarity of the founder haplotype. The last point is particularly important, because, unlike classical linkage methods where the information provided by dense SNP genotyping plateaus (e.g., updating a 100 K chip to a 500 K chip may not make much difference), for HRRA, a denser coverage increases sensitivity in detecting rare haplotypes shared among patients. The reason is that, while the traditional linkage analysis methods make use of familial data and therefore deal with recombination events in a few generations, HRRA tries to detect regions shaped by recombinations of tens of generations and does not rely on direct inheritance. There is reason to believe that typing the rare variants identified by next generation sequencing technology may further increase detection resolution to regions much smaller than 1 cM.

For complex diseases in a case–control scenario, it is possible that two or more individuals may have an unknown relationship and the shared ancestry may not necessarily have intrinsic connection with the underlying disease. This is unlikely the case and can be dealt with for the following reasons. First, close relationships (such as first and second degree relatives) should be identified and compared only to individuals who are not from the same family. Second, for distant relationships, the random chance of sharing any autosomal region IBD is low. For example, according to a previous simulation, the chance of sharing any region IBD on the autosomes is 1.6% for two 10th cousins and is 0.5% for three 6th cousins [Yang et al., 2008]. Most importantly, inheriting two copies IBD by one individual becomes much more unlikely to occur by chance than sharing a single haplotype. All considered, it is reasonable to conclude that HRRA preferentially detects the homozygous regions shared due to their intrinsic connection with the disease in question.

Without strong selection pressure against a recessive mutation that may not affect the early survival and reproduction of an individual, the mutation may persist in a population and affect individuals in a sporadic fashion. Therefore, the genetic cause of a recessive disease may not even be suspected in many cases, and more recessive mutations may exist than realized. The novel algorithm introduced in this study may lead to discoveries of unknown mutations of recent history, for both Mendelian diseases and in certain circumstances, complex diseases.
