# Supplementary material for: Homozygosity Mapping on a Single Patient—Identification of Homozygous Regions of Recent Common Ancestry by Using Population Data
Source: Hum Mutat. 2011 Jan 25;32(3):345–53. doi: 10.1002/humu.21432 (PMC3357498; doi:10.1002/humu.21432)
Supplement: Supplementary file 1 [file humu0032-0345-SD1.pdf]

Supporting Information for the article:

Zhang et al., ***Human Mutation***

Homozygosity mapping on a single patient--identification of homozygous regions of recent common ancestry by using population data

Lu Zhang[#], Wanling Yang[#*], Dingge Ying, Stacey S. Cherny, Friedhelm Hildebrandt,

Pak Chung Sham, and Yu Lung Lau

The program

The algorithm requires whole genome SNP genotyping data from patients, as well as genotyping data from a certain number of unrelated controls, ideally derived from the same population. For disease of Mendelian inheritance, it can be a single patient case, or multiple cases from different families. For complex diseases, patients can be either affected members from multiplex families or unrelated sporadic cases. The algorithm identifies homozygous regions in each individual genome that surpass a certain threshold (based on either physical size or a number of SNPs depending on the platform of genotyping used, and containing at least one SNP that is homozygous on a minor allele of certain allele frequency or below in order to filter out apparently common homozygous regions). Population allele frequency of the haplotype of the entire homozygous region was then estimated by a Markov model. The estimated allele frequency of this haplotype in the general population is then used to evaluate the probability of such a homozygous region to appear in one or more individual(s) by random chance through a simulation method. When this probability is extremely low, the haplotype can be assumed to have arisen from a recent common ancestry and is likely to harbor a disease-causal mutation.

Dealing with genotyping errors

To deal with the issue of interruption of continuous homozygous stretch by genotyping errors, HRRA allows certain percentage of genotyping errors and the threshold can be reset by users. For example, for an allowable error rate of 1%, if a heterozygous genotype is located in a stretch containing more than 99 homozygous genotypes and its distance with the heterozygous SNP nearest to it is more than 34 (>100/3) SNPs, this heterozygous genotype would be assumed to be caused by a genotyping error and would be converted to a homozygous genotype on the population major allele for further analysis.

Detecting the homozygous regions shared by multiple individuals

After selecting homozygous regions in all the individuals that surpass the selection criteria, HRRA then considers the regions shared among two or more individuals in the case group and the controls, respectively. The criteria for this selection for the simulation process described in the paper, a dataset based on 500,000 SNPs genotyped genome-wide, is when the selected homozygous regions overlap between two individuals for more than 10 SNPs in a case control setting and for affected sibpairs, and 1 SNP for single-gene disease scenario between patients from different families. The overlap had to be homozygous on the same allele assuming no

allelic heterogeneity. However, there is an option to assume allelic heterogeneity (among different patients but not on the same patient) that the overlapped region between individuals can be homozygous on different alleles.

The dataset used for simulation

Genotyping data on Chinese individuals collected locally in Hong Kong and genotyped on a platform of Illumina 610-Quad BeadChip was used to test the program. The data contains around half a million SNPs for each individual covering the entire genome. Quality control of the genotype data was performed to exclude certain SNPs with low call rate, low allele frequency and violation of Hardy-Weinberg equilibrium using PLINK[Purcell et al., 2007] and detailed information is available in our previous work[Yang et al., 2010].

The process of simulating homozygous regions with recent ancestral haplotypes

In each simulation, a mutation was randomly assigned to a SNP in the genome of a selected individual, serving as the founding common ancestor. Recombination process was then simulated through generations of meioses according to a method we reported previously[Yang et al., 2008]. Briefly, the recombination process was simulated according to a χ^2 model, which takes into consideration of physical interference on recombination but not chromatid interference. The genetic distance used for the crossover simulation process was adopted from HapMap, which reflects average male and female genetic distances for each chromosome. After simulation of the crossover process, which shortens the ancestral haplotype further and further by recombination events, the final “affected” individual was simulated to inherit two copies of the mutation allele, each being simulated independently through different paths of meioses. The haplotype of the relevant mutation-containing region was then imputed from the genotype of the afore-designated ancestral individual. Briefly, the first SNP at the leftmost of the region was determined by a randomized process based on the ancestral genotype and the population frequencies of it’s two alleles in random. The succeeding alleles were chosen according to a Markov model based on haplotype frequencies in controls with a randomized process.

For the simulation of case control data, the available individuals were randomly designated as “cases” and “controls”, and a number of "cases" were assigned to inherit two copies of a common ancestral allele simulated as described. For the simulation of affected sibships, both parents of affected sibpairs were simulated to inherit one allele of the mutation haplotype, and the “affected children” were simulated to inherit two copies of the mutation allele, one from each parent, with the other regions randomly inheriting an allele from the four-allele bundle from each parent after simulation of crossover events.

When evaluating shared homozygous regions for affected sib pairs, the region that produces the smaller HF^2 between the two siblings in each family was used for the calculation of HBC_m, a between-family parameter. Since the chance for a sibpair to share two alleles IBD is ¼, $(1/4)^N$ was also factored into the calculation of the P values afterwards, and N stands for the number of sibpairs who shared the same founder homozygous allele.

The Output and Graphical display of HRRA

The text output from HRRA includes all the regions ranked by $-\log(Pvalue)$, and the other information includes the physical position and the SNP ID on the edge of the homozygous region. For the graphical result, it includes the physical position and the $-\log(Pvalue)$ for each homozygous region, which is processed by an affiliated software GHRRA in the HRRA package for displaying the result graphically.

Computational efficiency

The program runs linear on the number of markers used, and because of the pair-wise comparisons for overlapped homozygous regions between individuals, it runs square on the total number of the individuals considered. The memory requirement is linear to both the sample size and the number of the SNPs. On a workstation PC machine with 4 processors the speed of 2.4 GHZ and 4 GB of memory, the program runs 3.3 hours for a single patient scenario with 1000 control individuals. It runs about 24 hours for a situation of 30 affected sibpairs with 1000 times of simulation to generate the control distribution curve, and about five days on case control scenarios with 200 cases also with 1000 simulations. It is worth noting that the computation time also depends on the genotype platform (number of SNPs) and the cutoff criteria used for initial selection of homozygous regions for processing.

The platform.

At present a windows version is available. Software that can work on other platforms is under development.

Calculation of the y-axis for the density distribution (gamma distribution) shown in Figure 1c,d and Figure 2d.

Calculation of the y-axis is based on the probability density function of gamma distributions. Briefly, the scale parameter θ and shape parameter k for gamma distribution of the feature (length or HF^2) of homozygous regions is estimated by Maximum Likelihood Estimation according to the following formula:

$$\hat{\theta} = \frac{1}{KN}\sum_{i=1}^{N} x_i$$

$$s = \ln\left(\frac{1}{N}\sum_{i=1}^{N} x_i\right) - \frac{1}{N}\sum_{i=1}^{N}\ln(x_i)$$

$$\hat{k} = \frac{3 - s + \sqrt{(S-3)^2 + 24s}}{12s}$$

$[x_1, x_2, ..., x_N]$ represented the values of the feature of homozygous regions from N times simulation. We then calculated the value of $f(x)$ based on the probability density function of a gamma distribution for every x_i, which is the value plotted on the y-axis.

$$f(x;k,\theta) = x^{k-1}\frac{e^{-x/\theta}}{\theta^k \Gamma(k)}$$

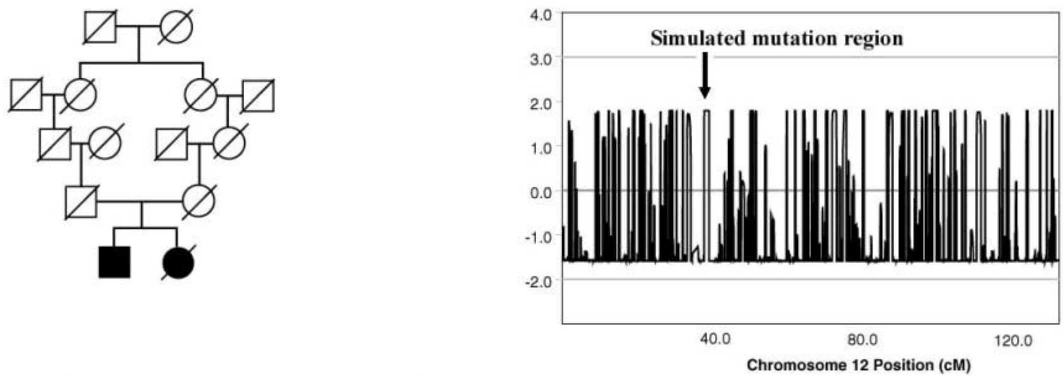

a An assumed family structure as input to Merlin **b** Merlin result on the case described in Figure 1b

Supp. Figure S1. Result from Merlin on a representative simulation described in Figure 1b. **a.** An assumed family structure served as an input to Merlin. Input to Merlin by assuming a second cousin marriage through parametric linkage by a recessive model (0.0001, 0.0001, 0.0001, and 1 for the model parameter file required by Merlin) and by adding a non-existent affected sibling with no genotyping information. **b.** Merlin result on the case described in Figure 1b. Shown is the Merlin result on Chromosome 12 where the simulated homozygous region is located. The Y-axis shows LOD scores from parametric linkage analysis on the recessive model.

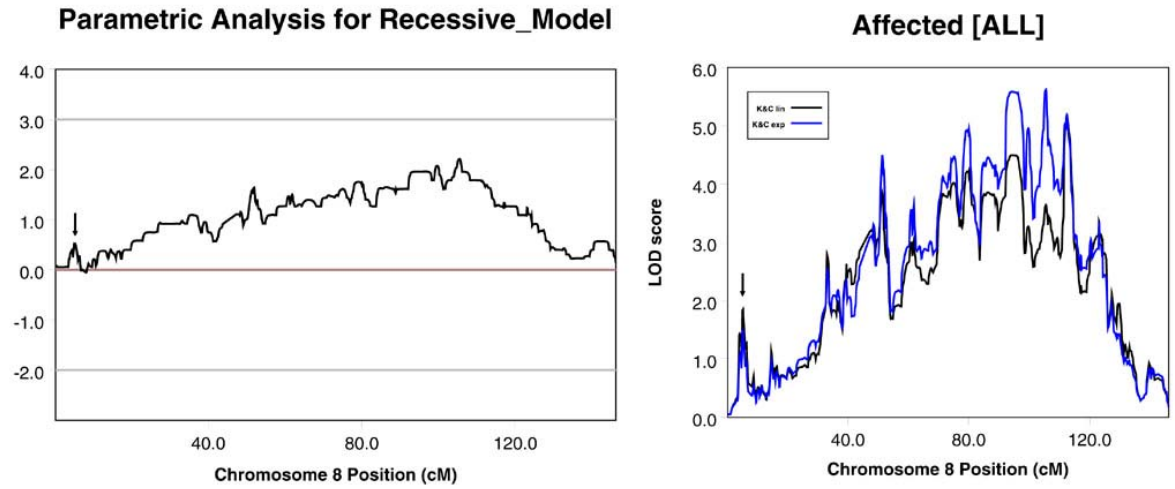

Merlin result on the same simulated case. Left: parametric; right, nonparametric

Supp. Figure S2. Merlin results on the sibpair case shown in Figure 4. Left panel: Result by parametric method on a recessive model (0.0001, 0.0001, 0.0001, 1 for the model file). Right panel: result from Merlin by nonparametric models (black curve is result from a linear model and the blue curve is result from an exponential model introduced by Kong and Cox [Kong and Cox, 1997]. Only 1 SNP in every 100Kbp region is used in these cases.
